# Supplementary material for: Assessing costs of Indonesian fires and the benefits of restoring peatland
Source: Nat Commun. 2021 Dec 2;12:7044. doi: 10.1038/s41467-021-27353-x (PMC8639972; doi:10.1038/s41467-021-27353-x)
Supplement: Supplementary file 1 — Supplementary Information [file 41467_2021_27353_MOESM1_ESM.pdf]

Supplement - Assessing costs of Indonesian fires and the benefits of restoring peatland

Kiely, L., Spracklen, D.V., Arnold, S. R., Papargyropoulou. E., Conibear, L., Wiedinmyer, C., Knote, C., Adrianto, H. A.

Section 1: Fire emissions

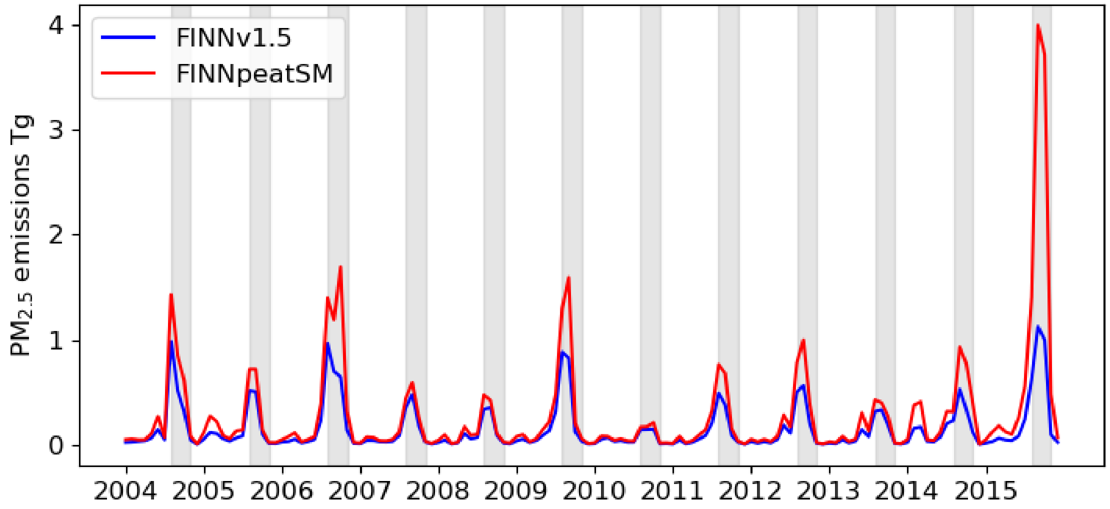

Supplementary Figure 1: $PM_{2.5}$ Monthly $PM_{2.5}$ emissions from FINNv1.5 and FINNpeatSM. The dry season (August-October) in each year is shaded.

Section 2: Costs included in analysis

Supplementary Table 1: Costs included in this study

**Category**		**Included in cost**	**Cost**	**Source**
CO_2 emissions		Average closing price of CO_2 in EU Emissions trading system during 2009-2020	\$11.8 tCO_2^{-1}	Business Insider [1]
Long term health impacts	DALY from $PM_{2.5}$ exposure	The economic loss of a DALY caused by non-communicable diseases.	\$4710 $DALY^{-1}$	World Economic Forum [2]<br>Mboi et al.[3]
Damage to Land cover	Oil palm	NPV of oil palm averaged over large scale, small scale high yield and low yield	\$8885 ha^{-1}	Butler et al.[4]; Noormahayu et al.[5]; Ruslandi et al.[6]; Fisher et al.[7]; Sofiyuddin et al.[8]; Svatonová et al.[9]; Sofiyuddin et al.[10]
	Crops	NPV of rice and maize	\$827 ha^{-1}	Sofiyuddin et al. [8]

	Wood fibre	NPV of Acacia plantations	\$1206 ha^{-1}	Sofiyuddin et al. [8]
	Rubber	NPV of rubber plantations	\$1662 ha^{-1}	Sofiyuddin et al. [8]<br>Sofiyuddin et al.[10]
	Logging	NPV of logging concessions	\$7713 ha^{-1}	Ruslandi et al.[6];<br>Fisher et al.[7];<br>Sofiyuddin et al. [8]
	Forest	Provisions of food, water, raw materials and medicinal resources.<br>Erosion prevention.<br>Genetic diversity and pollination services.<br>Cultural and recreational services.	\$4079 ha^{-1}	de Groot et al.[13]

<u>Section 3: Fire reduction in different PA types</u>

Table S2 shows the ratios of normalised burned area within and outside of protected areas. A value < 1 implies that there is less burned area per km^2 inside the protected areas than outside, a value > 1 suggests that there is more burned area per km^2. Some protected area types, such as Nature Reserves or Grand Forest Parks, seem to be less effective in reducing fires than others, such as National Parks. Of the 22 protected areas which contain peatland in Sumatra, 8 are Wildlife reserves and 12 are National Parks with only 1 Grand Forest Park and 1 Nature Recreation Park. In all years the burned area ratio is greater than 1 for the Grand Forest Park, suggesting that the protection against fires is low for this park. Table S3 shows the ratio of average soil moisture inside and outside of protected areas. The soil moisture is greater inside protected areas, where drainage canals are prohibited. Again, the difference is greatest for National Parks. A detailed description of the different protected area classifications is provided in Brun et al.[14].

Different protected area types have different average size. Grand Forest parks in Sumatra are 86 km^2 on average and Nature Reserves in Kalimantan are 469 km^2 on average. In contrast National Parks are much larger, on average 2826 km^2 in Sumatra and 4700 km^2 in Kalimantan. Table S4 shows the burned area ratios for protected areas of all categories over 1000 km^2 are mostly lower than for all protected areas over 100 km^2, showing that size is likely one factor in the level of protection from fire. This could be because large protected areas are less likely to be influenced by deforestation and drainage happening outside of the protected area. It is also possible that larger protected areas are more remote and are less effected by runaway fires from other land types.

Not all smaller protected areas have high burned area, however. Wildlife reserves are on average around 350 km^2, and these have substantially lower burned area ratios than Nature reserves. The differences here could be due to location or due to how well the protected area is managed. This could also explain the differences seen between protected areas in Sumatra and Kalimantan.

Supplementary Table 2: The average ratio of burned area per km^2 *on peatland within a protected areas to burned area per km^2 on peatland within 0.25° of the protected area for different years, split for different types of protected area and for Sumatra and Kalimantan. All protected areas included contain a mix of peatland and non-peatland, and the average ratio for non-peatland in National Parks is shown. The International Union for Conservation of Nature (IUCN) categories for the different types of protected areas are given.*

		2004		2006		2009		2012		2014		2015	
	IUCN	Suma	Kali	Suma	Kali	Suma	Kali	Suma	Kali	Suma	Kali	Suma	Kali
National Parks	II	0.28	0.30	3.69	0.41	0.60	0.21	0.37	0.33	0.58	0.23	1.17	0.63
(Non-peat)		0.90	0.16	0.35	0.22	0.89	0.12	0.53	0.06	0.81	0.09	0.68	0.43
Grand Forest Park	V	1.39	-	2.45	-	1.97	-	1.35	-	1.54	-	2.47	-
Nature Recreation Park	V	-	-	0.54	0.80	0.55	-	0.37	4.07	0.71	2.35	0.68	4.09
Nature Reserve	Ia	-	1.15	-	0.92	-	1.97	-	1.37	-	1.33	-	0.93
Wildlife Reserve	IV	0.71	0.34	0.29	0.54	0.19	0.56	0.82	0.32	0.16	0.12	0.28	0.58
Undesignated		-	2.18	-	0.89	-	1.55	-	0.98	-	1.09	-	2.00

Supplementary Table 3: The average ratio of average soil moisture on peatland inside a protected are to average soil moisture in peatland outside a protected area (within 0.25° of the protected area) for August, September and October 2015. The average ratio is shown for protected areas in Sumatra and Kalimantan separately, and for all protected areas and National parks only.

	August		September		October	
	Suma	Kali	Suma	Kali	Suma	Kali
National Parks	1.34	1.48	1.41	1.57	1.35	1.49
All protected areas	1.12	1.35	1.13	1.44	1.11	1.33

Supplementary Table 4: The average ratio of burned area per km^2 on peatland within a protected areas to burned area per km^2 on peatland within 0.25° of the protected area for different years and for Sumatra and Kalimantan. The average over all protected areas over 100 km^2 and for all protected areas over 1000 km^2 is shown.

PA type	2004		2006		2009		2012		2014		2015	
	Suma	Kali	Suma	Kali	Suma	Kali	Suma	Kali	Suma	Kali	Suma	Kali
>100 km^2	0.58	0.54	1.89	0.74	0.75	0.68	0.72	0.74	0.65	0.71	0.86	1.23
>1000 km^2	0.25	0.39	0.18	0.56	1.00	0.32	0.48	0.41	1.02	0.33	0.96	0.82

Section 4: Contribution of Non-Indonesian Fires to health impacts

The simulated $PM_{2.5}$ used to estimate health impacts of fires is from all fire emissions in the study area, not only from Indonesian fire emissions. For 2015 Indonesian fires produced 97% of the total PM2.5 emissions in the study area, with the remaining coming from fires in Malaysia, Brunei and

mainland South-East Asia. In other years non-Indonesian fires contributed 4-7% of total $PM_{2.5}$ emissions.

For 2015 we have also found the $PM_{2.5}$ concentrations and health impacts resulting from Indonesian fires only. These non-Indonesian fires resulted in 1900 mortalities (3% of the mortalities from all fires) and 76,500 DALYs (3% of DALYs from all fires). For the mortalities in Singapore non-Indonesian fires resulted in 4% of the mortality and DALYs caused by all fires. For Indonesian and Malaysian mortalities it was 3%. In other years the health impact contributions are likely to be similar.

The costs resulting from CO_2 emissions and damage to land cover are calculated from Indonesian fires only, while the costs relating to health impacts are from all fires. These non-Indonesian fires could be contributing 3-7% to the health impacts.

Section 5: Estimating DALYs

In Kiely et al.[15] it is shown that the $PM_{2.5}$ exposure and premature mortality caused by fires has a strong linear relationship with the total $PM_{2.5}$ emissions and resulting SOA. In Figure S2 we show that this linear relationship also holds for DALYs from the study. Using 0.16 million DALYs per Tg $PM_{2.5}$ and SOA, we estimate the reduction in DALYs resulting from peatland restoration, shown in table S5. For 2015 the reduction in $PM_{2.5}$ emissions and resulting SOA after 2.49Mha of peatland have been restored is 2.99 Tg. With the relationship in Figure S2, this suggests a reduction of 0.48 million DALYs. Using the simulated $PM_{2.5}$ for 2015 with peatland restoration, and the health impact equations, the total DALYs estimated to result from fires in 2015 after 2.49 Mha of peatland has been restored is 1.72 million giving a reduction of 0.47 million DALYs, close to the estimate using the $PM_{2.5}$ emissions relationship (1.71 million).

In order to separate these DALYs into the countries effected, we use the percentage of total DALYs from Indonesia, Malaysia and Singapore from Kiely et al.[15]. 54%, 55%, 47%, 52%, 55% and 58% of the total DALYs come from Indonesia in 2004, 2006, 2009, 2012, 2014 and 2015 respectively. From Malaysia it is 13%-16% for these years and for Singapore is 3.1% - 4.0%.

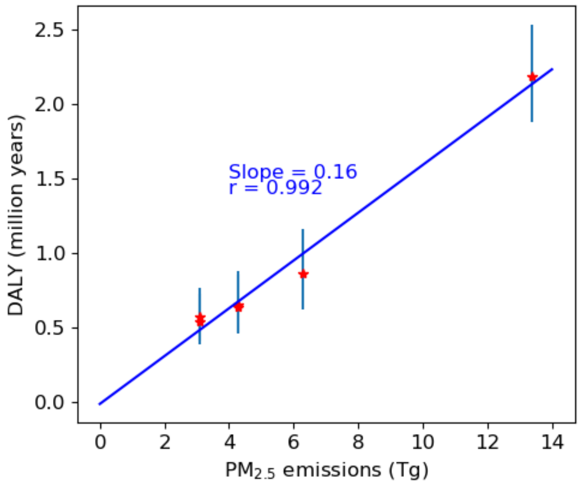

Supplementary Figure 2: Estimated DALYs resulting from exposure to $PM_{2.5}$ from August – October fires in 2004, 2006, 2009, 2012, 2014 and 2015, as a function of total August-October $PM_{2.5}$ emissions and resulting SOA. All data from Kiely et al.[15]*. The upper and lower 95% uncertainty interval centred at the mean is shown for the DALYs. A line of best fit is shown with the gradient of the line and the r correlation shown on the plot.*

Supplementary Tables 5: The reduction in $PM_{2.5}$ and SOA from restoring 2.49 Mha of peatland to the level of National Parks, and the DALYs and premature mortality before and after this restoration. Health impacts before restoration are calculated from the simulated $PM_{2.5}$ concentrations and health impact equations, the reduction in health impacts is calculated using the relationship of 0.16 million DALYs per Tg fire emissions, the reduction in mortality is based on 4110 deaths per Tg reduction in PM emission.

Year	Reduction in $PM_{2.5}$ and SOA (Tg)	DALY without restoration	DALY after restoration	Mortality without restoration	Mortality after restoration
2004	0.41	637,727	572,127	16,219	14,533
2006	0.11	867,220	849,620	22,088	21,635
2009	0.59	654,733	560,973	16,656	14,231
2012	0.33	573,084	520,124	14,573	13,216
2014	0.41	541,086	475,166	13,705	12,019
2015	2.99	2,187,614	1,709,374	55,819	43,530

References

1. Business Insider. CO2 EUROPEAN EMISSION ALLOWANCES IN USD – HISTORICAL PRICES. *Markets Insider* (2020). Available at: https://markets.businessinsider.com/commodities/historical-prices/co2-european-emission-allowances/euro/23.4.2006_23.5.2020.
2. World Economic Forum. *Economics of Diseases in Indonesia*. (2015).
3. Mboi, N. *et al.* On the road to universal health care in Indonesia, 1990–2016: a systematic analysis for the Global Burden of Disease Study 2016. *Lancet* **392,** 581–591 (2018).
4. Butler, R. A., Koh, L. P. & Ghazoul, J. REDD in the red: palm oil could undermine carbon payment schemes. *Conserv. Lett.* **2,** 67–73 (2009).
5. Noormahayu, M. N., Khalid, A. R. & Elsadig, M. A. Financial assessment of oil palm cultivation on peatland in Selangor, Malaysia. **5,** 1–18 (2009).
6. Ruslandi, Venter, O. & Putz, F. E. Overestimating conservation costs in Southeast Asia. *Front. Ecol. Environ.* **9,** 542–544 (2011).
7. Fisher, B., Edwards, D. P., Giam, X. & Wilcove, D. S. The high costs of conserving Southeast Asia's lowland rainforests. *Front. Ecol. Environ.* **9,** 329–334 (2011).
8. Sofiyuddin, M., Rahmanulloh, A. & Suyanto, S. Assessment of Profitability of Land Use Systems in Tanjung Jabung Barat District, Jambi Province, Indonesia. *Open J. For.* **02,** 252–256 (2012).
9. Svatonová, T., Herák, D. & Kabutey, A. Financial profitability and sensitivity analysis of palm oil plantation in Indonesia. *Acta Univ. Agric. Silvic. Mendelianae Brun.* **63,** 1365–1373 (2015).
10. Sofiyuddin, M., Suyanto, S., Kadir, S. & Dewi, S. Sustainable land preparation for farmer-managed lowland agriculture in Indonesia. *For. Policy Econ.* **130,** 102534 (2021).
11. Sofiyuddin, M., Rahmanulloh, A. & Suyanto, S. Assessment of Profitability of Land Use Systems in Tanjung Jabung Barat District, Jambi Province, Indonesia. *Open J. For.* **02,** 252–256 (2012).

12. de Groot, R. *et al.* Global estimates of the value of ecosystems and their services in monetary units. *Ecosyst. Serv.* **1,** 50–61 (2012).
13. de Groot, R. *et al.* Global estimates of the value of ecosystems and their services in monetary units _ Elsevier Enhanced Reader.pdf. 50–61 (2012).
14. Brun, C. *et al.* Analysis of deforestation and protected area effectiveness in Indonesia: A comparison of Bayesian spatial models. *Glob. Environ. Chang.* **31,** 285–295 (2015).
15. Kiely, L. *et al.* Air quality and health impacts of vegetation and peat fires in Equatorial Asia during 2004 – 2015. *Environ. Res. Lett.* **15,** (2020).
