# Supplementary material for: Assessing costs of Indonesian fires and the benefits of restoring peatland
Source: Nat Commun. 2021 Dec 2;12:7044. doi: 10.1038/s41467-021-27353-x (PMC8639972; doi:10.1038/s41467-021-27353-x)
Supplement: Supplementary file 3 — Reporting Summary [file 41467_2021_27353_MOESM3_ESM.pdf]

nature portfolio

Corresponding author(s): Laura Kiely

Last updated by author(s): Oct 3, 2021

Reporting Summary

Nature Portfolio wishes to improve the reproducibility of the work that we publish. This form provides structure for consistency and transparency in reporting. For further information on Nature Portfolio policies, see our Editorial Policies and the Editorial Policy Checklist.

Statistics

For all statistical analyses, confirm that the following items are present in the figure legend, table legend, main text, or Methods section.

n/a	Confirmed	
☐	☒	The exact sample size (n) for each experimental group/condition, given as a discrete number and unit of measurement
☒	☐	A statement on whether measurements were taken from distinct samples or whether the same sample was measured repeatedly
☒	☐	The statistical test(s) used AND whether they are one- or two-sided<br>*Only common tests should be described solely by name; describe more complex techniques in the Methods section.*
☒	☐	A description of all covariates tested
☐	☒	A description of any assumptions or corrections, such as tests of normality and adjustment for multiple comparisons
☐	☒	A full description of the statistical parameters including central tendency (e.g. means) or other basic estimates (e.g. regression coefficient) AND variation (e.g. standard deviation) or associated estimates of uncertainty (e.g. confidence intervals)
☒	☐	For null hypothesis testing, the test statistic (e.g. F, t, r) with confidence intervals, effect sizes, degrees of freedom and P value noted<br>*Give P values as exact values whenever suitable.*
☒	☐	For Bayesian analysis, information on the choice of priors and Markov chain Monte Carlo settings
☒	☐	For hierarchical and complex designs, identification of the appropriate level for tests and full reporting of outcomes
☒	☐	Estimates of effect sizes (e.g. Cohen's d, Pearson's r), indicating how they were calculated

Our web collection on statistics for biologists contains articles on many of the points above.

Software and code

Policy information about availability of computer code

Data collection	WRF-chem version 3.7.1 was used to create model data. This is available from https://www2.mmm.ucar.edu/wrf/users/download/get_sources.html#WRF-Chem.
Data analysis	All data analysis was done using python code written for the study, using Python version 2.7 .

For manuscripts utilizing custom algorithms or software that are central to the research but not yet described in published literature, software must be made available to editors and reviewers. We strongly encourage code deposition in a community repository (e.g. GitHub). See the Nature Portfolio guidelines for submitting code & software for further information.

Data

Policy information about availability of data

All manuscripts must include a data availability statement. This statement should provide the following information, where applicable:

- Accession codes, unique identifiers, or web links for publicly available datasets
- A description of any restrictions on data availability
- For clinical datasets or third party data, please ensure that the statement adheres to our policy

The fire emissions and burned area data used in and generated for this study can be accessed from NERC EDS Environmental Information Data Centre at https://doi.org/10.5285/fdae44ed-8b22-4935-b889-b4b271138385. The Transparent World tree plantations data used in this study can be accessed from Global Forest Watch at https://data.globalforestwatch.org/datasets/gfw::tree-plantations/about. The European Space Agency land cover CCI data used in this study can be accessed from www.esa-landcover-cci.org/?q=node/164. The logging concessions data from the Ministry of Environment and Forestry Indonesia used in this study can be accessed from http://geoportal.menlhk.go.id/arcgis/rest/services/KLHK. The World Resources Institute Peat lands data used in this study can be accessed from Global Forest Watch at https://data.globalforestwatch.org/datasets/gfw::indonesia-peat-lands/about. The World Database on Protected Areas (WDPA) from

the United Nations Environment Programme (UNEP) and the International Union for Conservation of Nature (IUCN) can be accessed from Protected Planet at www.protectedplanet.net/en.

Field-specific reporting

Please select the one below that is the best fit for your research. If you are not sure, read the appropriate sections before making your selection.

☐ Life sciences ☐ Behavioural & social sciences ☒ Ecological, evolutionary & environmental sciences

For a reference copy of the document with all sections, see nature.com/documents/nr-reporting-summary-flat.pdf

Ecological, evolutionary & environmental sciences study design

All studies must disclose on these points even when the disclosure is negative.

Study description	We used remote sensed data on emissions from landscape fires in Indonesia combined with data from the WRF-chem regional chemistry-climate model to explore the impacts of air pollution from fires.
Research sample	We used data on emissions from landscape fires available from the FINNpeatSM dataset. This dataset is described in full in Kiely et al. (2019) which is available and fully open access at https://acp.copernicus.org/articles/19/11105/2019/acp-19-11105-2019.html. The domain was chosen over the region of interest, covering Indonesia.
Sampling strategy	All data over the domain was analysed, and there was no sample size calculation.
Data collection	Data from pre-existing datasets was used. The WRF-chem regional chemistry-climate model was used to simulate air pollution over the domain of interest. The model is open access and available for download. No other primary data was collected during this study.
Timing and spatial scale	Data was analysed for the period covering 2004 to 2015. Remote sensed data of emissions from landscape fires was available at 1km by 1 km spatial scale. Modelling data on air pollution was simulated at 30 km by 30 km spatial scale.
Data exclusions	No data were excluded.
Reproducibility	All data in this study is from analysis of existing remote sensed datasets and from numerical models and are reproducable. All attempts at replication, including simulations of multiple years, were successful.
Randomization	Not relevant for our modelling study
Blinding	Not relevant for our modelling study

Did the study involve field work? ☐ Yes ☒ No

Reporting for specific materials, systems and methods

We require information from authors about some types of materials, experimental systems and methods used in many studies. Here, indicate whether each material, system or method listed is relevant to your study. If you are not sure if a list item applies to your research, read the appropriate section before selecting a response.

Materials & experimental systems

n/a	Involved in the study	
☒	☐	Antibodies
☒	☐	Eukaryotic cell lines
☒	☐	Palaeontology and archaeology
☒	☐	Animals and other organisms
☒	☐	Human research participants
☒	☐	Clinical data
☒	☐	Dual use research of concern

Methods

n/a	Involved in the study	
☒	☐	ChIP-seq
☒	☐	Flow cytometry
☒	☐	MRI-based neuroimaging
